# High-Dose Cyclophosphamide Administration Orchestrates Phenotypic and Functional Alterations of Immature Dendritic Cells and Regulates Th Cell Polarization

**DOI:** 10.3389/fphar.2020.00775

**Published:** 2020-05-25

**Authors:** Lei Bao, Changfu Hao, Juan Wang, Di Wang, Youliang Zhao, Yiping Li, Wu Yao

**Affiliations:** ^1^Department of Occupational Health and Environmental Health, School of Public Health, Hebei Medical University, Shijiazhuang, China; ^2^Department of Occupational Health and Environmental Health, School of Public Health, Zhengzhou University, Zhengzhou, China; ^3^Hebei Key Laboratory of Environment and Human Health, Hebei Medical University, Shijiazhuang, China; ^4^Department of Statistics, Hebei General Hospital, Shijiazhuang, China

**Keywords:** cyclophosphamide, immature dendritic cells, antigen uptake capacity, T cell priming, Th polarization

## Abstract

High-dose cyclophosphamide (CTX) inhibits the immune response. Dendritic cells (DCs) are professional antigen presenting cells (APCs) with a crucial role in initiating immune responses and sustaining immune tolerance. The relative contribution of DCs to immunosuppression induced by high-dose CTX is not well-documented. In this study, we employed the CTX-induced immunosuppressive rat model to examine alterations in DCs. We generated and cultured monocyte-derived immature DCs (imDCs) *in vitro* and explored their capacity of antigen uptake, T cell priming, cytokine production, and surface marker expression following high-dose CTX. Subsequently, we co-cultured CTX-treated imDCs with Th cells to determine Th cell polarization, and further explored the Toll-like receptor/Myeloid differentiation primary response 88/Mitogen-activated protein kinase (TLR/MyD88/MAPK) pathway. Our results show reduced cell number and surface maker alterations in splenic CD103^+^ DCs of CTX-treated immunosuppressed rats. *In vitro*, high-dose CTX weakened the antigen uptake capacity and enhanced the T cell priming capacity of imDCs, in addition to triggering imDC surface marker alterations. TLR, MyD88, and MAPK expression levels, involved in mediating Th cell polarization, were also significantly elevated. Our collective findings indicate that high-dose CTX administration potentiates phenotypic and functional alterations of imDC. Such changes may contribute to the regulation of Th polarization.

## Introduction

Recent years have witnessed a rapid rise in immunosuppressive diseases, due to factors such as increased environmental pollution, ecological imbalance, and the diversity of new chemicals. Research on immunosuppressants is therefore particularly important and urgently needed. Cyclophosphamide (CTX), the most widely used immunosuppressant, is hydrolyzed into phosphoramide mustard that affects all stages of immunity by inhibiting or killing immune cells directly (Agus et al., 1986; Kurauchi et al., 2017). However, the mechanism remains unclear, and the majority of past CTX-relevant investigations appear to have overlooked the effect of CTX on dendritic cells (DCs). DCs are critical antigen-presenting cells (APCs) that can initiate immune responses and induce immune tolerance, as well as determine the direction of Th polarization (Esterhazy et al., 2016; de Lastic et al., 2016). Therefore, a systemic study of the role of CTX on DCs is required.

The majority of DCs *in vivo* exist in an immature state, designated as immature DC (imDC), and exhibit high antigen uptake capacity (Wilson et al., 2004). ImDCs can recognize multiple pathogen-associated molecular patterns (PAMPs) through pattern recognition receptors (PRRs), such as lipopolysaccharide (LPS), GpG-DNA, peptidoglycan, lipoprotein, and mycobacterial cell wall components (Wilbers et al., 2016; Qian and Cao, 2018). In addition, only imDCs can mediate immune tolerance *via* the induction of T cell apoptosis and regulatory T (Treg) cell formation (Dudek et al., 2013; McGovern et al., 2017; Waisman et al., 2017). Following recognition of PAMPs, imDCs elevate their antigen presentation ability and undergo maturation by increasing the expression of MHC-like and costimulatory molecules. Meanwhile, mature DCs (mDCs) have the ability to initiate specific immune responses and regulate helper T (Th) cell polarization (Chow et al., 2016; Eisenbarth, 2019).

CTX is inactive *in vitro*, and is hydrolyzed into aldehyde phosphoramide by hepatic cytochrome P450 enzymes including CYP2B6, CYP3A4, and CYP3A5 *in vivo*, resulting in the formation of the toxic chemical phosphoramide mustard (Ekhart et al., 2008; Cartin-Ceba et al., 2016). Therefore, current studies have predominantly examined the effects of CTX *in vivo* (Salem et al., 2009; Salem et al., 2010; Weir et al., 2014). However, the results derived through this approach may be affected by both the *in vivo* environment and the cytokine milieu. Recent studies indicating that the expression of P450 family members including CYP1A1 and CYP1B1, could be elevated in bone marrow-derived DCs in response to PM_2.5_ (Castañeda et al., 2018) and aflatoxin (AF) B_1_ (Mehrzad et al., 2018), suggests that DCs also have metabolic capacity *in vitro*. Low-dose CTX enhances the anti-tumor immune responses by augmenting DC-mediated antigen presentation and cytokine secretion, which could increase the expansion of Th1 cells and attenuate the suppressor function of Tregs (Radojcic et al., 2010; Son et al., 2015). In addition, an increase in IL-17-producing CD4^+^ T cells, indicative of Th17 polarization, has also been observed following low-dose CTX (Madondo et al., 2016). However, few studies have focused on the effects of high-dose CTX administration on DCs, and the subsequent DC-mediated Th polarization.

In the present study, we aimed to examine the potential impact of high-dose CTX on DCs. Firstly, we examined the effect of CTX on CD103^+^ DCs in an experimental immunosuppressive rat model. Secondly, we focused on the peripheral blood-derived imDC-associated: i) functions; ii) surface marker expression; and iii) the polarization patterns of Th cells *in vitro*. Finally, we evaluated the expression patterns of Toll-like receptor/Myeloid differentiation primary response 88/Mitogen-activated protein kinase (TLR/MyD88/MAPK) proteins, with a view to elucidate the mechanisms underlying the DC-mediated regulation of Th cell polarization in response to high-dose CTX.

## Materials and Methods

### Experimental Animals

Male Sprague-Dawley (SD) rats (6–8 weeks, 180–220 g) were purchased from the Laboratory Animal Center of Henan Province (Zhengzhou, China). All rats were maintained in a specific pathogen-free (SPF) laboratory animal facility of Zhengzhou University. Cages, bedding, and food were sterilized by autoclaving. All experimental procedures were performed in strict accordance with the National Institute of Health Guide for Care and Use of Laboratory Animals (NIH Publication no. 8203) and approved by the Institutional Animal Care and Use Committee of Zhengzhou University.

### Human Subjects

Whole blood products (buffy coats) from healthy, anonymous donors were purchased from the Henan Red Cross Blood Center (Zhengzhou, China), after obtaining informed consent. All experimental procedures were performed in strict accordance with the ethical requirements of biomedical research issued by the international and national organization, and approved by the Ethics Committee of Zhengzhou University.

### CTX Treatment *In Vivo*

CTX (Sigma-Aldrich, St. Louis, USA) was dissolved in phosphate buffered saline (PBS) and gavaged at a dose of 15 mg/kg to the rats for 28 days. The control group was gavaged with identical volumes of PBS in the same manner. Rats were anesthetized with excessive carbon dioxide.

### CTX Treatment *In Vitro*

In DC viability measurements, propidium iodide (PI) staining was used to exclude dead cells from analysis. After 24 h of CTX treatment, DCs (5 × 105) were collected *via* centrifugation (300 × g, 5 min) and resuspended with 20 μl PI solution. The ratio of living to total acquired cells was used to calculate cell viability. CV75, the CTX concentration that resulted in 75% DC viability (25% cytotoxicity), was calculated by log-linear interpolation.

### Generation of imDCs

Peripheral blood mononuclear cells (PBMCs) were isolated using the Ficoll-Paque method (GE Healthcare Life Sciences, Piscataway, NJ) from buffy coats. CD14^+^ monocytes were isolated from PBMCs using MidiMACS Technology with CD14 MicroBeads (Miltenyi Biotec, Bergisch Gladbach, Germany). Next, CD14^+^ monocytes were cultured at 1 × 106 cells/ml in Roswell Park Memorial Institute (RPMI)-1640 medium (Gibco, Grand Island, NY, USA) supplemented with 10% fetal bovine serum (FBS; Gibco) in the presence of GM-CSF and IL-4 (50 ng/ml and 35 ng/ml; R&D Systems, Minneapolis, MN, USA) at 37°C and 5% CO2 for 7 days. On day 3, half of the medium was removed from culture and replenished with the same volume of fresh medium containing twofold concentrations of GM-CSF and IL-4. On day 5, the same step was repeated. On day 7, the imDCs were ready for experimental use.

### Flow Cytometric Analysis of Th Cells

Detection of Th cells in the peripheral blood of rats was performed according to the literature (Lei et al., 2018).

### Histological Analysis and CD103^+^DCs Immunofluorescence

The spleen samples were fixed in 4% paraformaldehyde (PFA), embedded in paraffin, and sectioned for staining with hematoxylin and eosin (H&E) staining to assess the degree of immunosuppression. Immunofluorescence (IF) was performed as follows. The same sections of spleen were fixed in 10% neutral formalin and embedded in paraffin. Next, paraffin sections were deparaffinized, rehydrated in xylene and ethanol, and treated with 3% H_2_O_2_ for 10 min. After heating in citrate butter for 20 min, sections were blocked with 10% goat serum in Tris-buffered saline (TBS) for 1 h at room temperature. Subsequently, sections were incubated overnight at 4°C with rabbit anti-rat CD103 (dilution 1:200; Abcam). After washing with PBS, sections were incubated with fluorescein isothiocyanate (FITC) goat anti-mouse IgG (dilution 1:400, Boster Biological Technology, Wuhan, China) for 1 h. 4′,6-diamidino-2-phenylindole (DAPI) was added for 10 min followed by three washes with PBS. Each slice was randomly selected from five visual fields, and Image-Pro Plus 6.0 software (Media Cybernetics, Silver Spring, Maryland, USA) used to analyze the positive cells in individual images.

### Magnetic Separation of Splenic CD103^+^ DCs

A portion of the rat spleen was minced and incubated in 5 ml RPMI-1640 (Gibco) with 2 mg/ml collagenase D (Roche Diagnostics GmbH, Mannheim, Germany) for 25 min at 37°C, followed by the addition of 10 mM ethylenediaminetetraacetic acid (EDTA) and incubation for 5 min. After digestion, splenic cells were dispersed by gentle pipetting, filtered through a 75 μm cell strainer, and used to prepare a single cell suspension. Cells were isolated using anti-CD103 microbeads (Miltenyi Biotec) and the MidiMACS Starting Kit (Miltenyi Biotec). The labeled cells were identified as CD103^+^ DCs.

### Flow Cytometric Analysis of CD103^+^ DCs and imDC Surface Markers

Isolated CD103^+^ DCs (1 × 10^6^) were subjected to flow cytometric analysis. Mouse-anti-rat CD54-Alexa Fluor^®^488 (R&D Systems), CD86-phycoerythrin (PE)-Vio770 (Miltenyi Biotec), CD80-PE (eBioscience, San Diego, CA, USA), CD11b/c-allophycocyanin (APC; Biolegend, San Diego, CA, USA), RT1B (MHC- II)-FITC (BD Biosciences, San Jose, CA, USA), CD1d-APC (eBioscience), and the respective matched isotype controls were employed.

The following markers were used for the staining of imDCs (1 × 10^6^): mouse-anti-human CD80-FITC, CD54-PE, CD86-peridinin chlorophyll (PerCP)-Cy™5.5, MHC-II-APC, CD1a-FITC, CD83-PE, CD11c-PerCP-Cy™5.5, and the respective matched isotype controls (BD Biosciences). All samples were washed, resuspended in 2% PFA, and subjected to flow cytometric analysis (Accuri C6; BD Biosciences).

### Antigen Uptake Capacity of imDCs

To assess pinocytosis capacity, imDCs (1 × 105) were incubated with 1 mg/ml FITC-labeled dextran (Sigma-Aldrich) for 1 h. A control sample was simultaneously incubated on ice. All samples were kept on ice until flow cytometric analysis was performed. Uptake of FITC-dextran was determined by measuring the mean fluorescence intensity (MFI) of FITC by flow cytometry. Dead cells were excluded from analysis based on PI fluorescence (BD Biosciences).

### Mixed Lymphocyte Reaction (MLR) to Examine the T Cell Priming Capacity of imDCs

To assess the T cell priming capacity of CTX-treated imDCs, a one-way MLR was performed. CD4^+^ T cells (2 × 106) isolated from PBMCs using CD4 MicroBeads (Miltenyi Biotec), were labeled with 10 μM carboxyfluorescein succinimidyl ester (CFSE; Invitrogen, Carlsbad, CA, USA) for 20 min at 37°C and 5% CO2. Subsequently, CD4^+^ T cells were suspended in RPMI-1640 medium containing 10% FBS at a density of 5 × 105 cells/ml, before the addition of the CFSE (1:1,000 dilution) staining. Immature DCs were centrifuged (300 × g, 5 min) and resuspended in RPMI-1640 medium at a density of 2.5 × 105 cells/ml. Finally, CFSE-labeled CD4^+^ T cells (100 µl) were incubated with 100 µl CTX-treated imDCs in RPMI-1640 medium for 7 days at 37°C and 5% CO2. The proliferation of CD4^+^ T cells was determined by measuring CFSE fluorescence by flow cytometry.

### Enzyme-Linked Immunosorbent Assay (ELISA) to Measure Cytokine Production by imDCs

To measure cytokine production by CTX-treated imDCs (1 × 106), acellular supernatants were collected and analyzed for IL-1β, IL-4, IL-6, IL-12p70, transforming growth factor (TGF)-β1, and IFN-γ levels using commercial ELISA kits, according to the manufacturer's protocol (Abconal, Wuhan, CN, USA). Plates were read using an ELISA plate reader (Bio-Tek ELx808, Winooski, VT, USA) at 450 nm.

### Quantitative Analysis of imDC Gene Expression

Genetic sequences for the following genes: TLR2, TLR4, TLR9, MyD88, T-bet, GATA-3, FoxP3, and RORγt were obtained from GenBank (National Center for Biotechnology Information, NCBI; **Table S1**) and the primers were custom-made (Sangon Biotec, Shanghai, China). Isolation of total RNA was performed aseptically on CTX-treated imDCs (1 × 106; for TLR2, TLR4, TLR9, and MyD88) and CD4^+^ T cells co-cultured with CTX-treated imDCs for 24 h at a CD4^+^ T cells to imDCs ratio of 2:1 (for T-bet, GATA-3, FoxP3, and RORγt), using the TRI Reagent procedure (Sigma-Aldrich), according to the manufacturer's protocol. Reverse transcription was performed in a total reaction volume of 20 µl, containing 2 µg total RNA, 10 μM random primers, 2.5 nM dNTPs, 0.1 M dithiothreitol (DTT), 20 U RNaseOUT (Invitrogen Waltham, MA, USA), and RNase-free water, at 37°C for 15 min and 85°C for 5 s. Real-time quantitative (RT-q) PCR was performed in a total volume of 25 µl containing 12.5 µl SYBR Premix Ex Taq II, 1.0 µl each of forward and reverse primers (10 µM), 2 µl cDNA, and 8.5 µl dH2O, using the PrimeScriptTM RT reagent Kit (Takara, Dalian, China). The optimized thermal cycling conditions were as follows: 95°C for 30 s, 40 cycles of 95°C for 5 s, and 60°C for 60 s. RT-qPCR was performed using a StepOnePlus Real-Time PCR system (Applied Biosystems, Foster City, CA, USA).

### Western Blotting to Measure ERK, JNK, and p38 Kinase Activation

CTX-treated imDCs (1 × 106) were lysed in radioimmunoprecipitation assay (RIPA) lysis buffer (Beyotime, Haimen, China) containing protease inhibitors (Beyotime) and phosphatase inhibitors (Roche, Basel, Switzerland). Lysates were separated on 10% SDS polyacrylamide gels and transferred onto polyvinylidene difluoride membranes (Millipore). Membranes were blocked with 5% milk for 1 h and incubated overnight at 4°C with primary anti-ERK, anti-phospho-ERK, anti-JNK, anti-phospho-JNK, anti-p38, anti-phospho-p38, and anti-β-Actin antibodies (dilution 1:1,000, Cell Signaling Technology, Boston, MA, USA), followed by horseradish peroxidase-conjugated anti-rabbit or anti-mouse IgG (Cell Signaling Technology) for 30 min at 37°C. Immunoreactive bands were visualized using an enhanced chemiluminescence system (ECL; GE Healthcare, Madrid, Spain).

### Statistical Analysis

All data were analyzed using SPSS version 17.0 software (SPSS Inc., Chicago, IL, USA). Continuous variables were expressed as mean ± standard error of the mean (SEM). To determine differences between variables, one-way analysis of variance followed by a pair-wise comparison with Student-Newman-Keuls test was performed. P values <0.05 were considered statistically significant, unless otherwise indicated.

## Results

### High-Dose CTX Caused Immunosuppression in Rats

**Figure 1:**
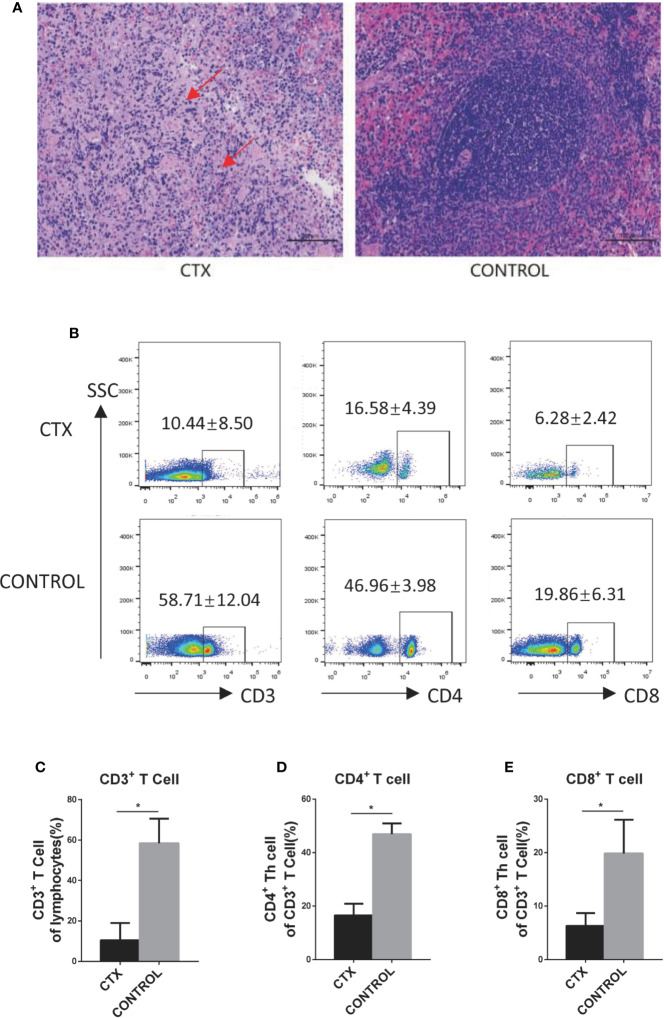
High-dose CTX caused immunosuppression in rats. **(A)** H&E staining of the CTX-induced pathological changes in the spleen of rats (200×). **(B)** High-dose CTX induced a reduction in the percentage of CD3^+^, CD4^+^, and CD8^+^ T cells in the peripheral blood of rats. The quantitative results of CD3^+^ **(C)**, CD4^+^ **(D)**, and CD8^+^ **(E)** T cells in the peripheral blood. n = 6. Error bars indicate SEM. *P < 0.05, compared with the control group. All values represent the mean ± SEM.

First, we established a CTX-induced immunosuppressive rat model to accurately determine the effects of high-dose CTX on DCs *in vivo*. Light microscopy (using a Nikon Eclipse Ci instrument) revealed spleen nodule structures disappeared, red and white pulp structures were disordered, and a large amount of fibrosis was evident in the connective tissues in the CTX-treated group (**Figure 1A**). Similar changes in the spleen have been previously demonstrated (Duggina et al., 2015; Lapointe et al., 2016). Next, we demonstrated that, compared to the control group, the body weight, spleen, and thymus coefficients were markedly reduced in the CTX-treated group (*P* < 0.05; **Table S2**). The percentages of CD3^+^, CD4^+^, and CD8^+^ T cells in peripheral blood were significant reduced in CTX-treated rats, relative to the control group (*P* < 0.05; **Figures 1B–E**). Evidences of those were clearly indicative of immunosuppression in our rat model.

### High-Dose CTX Altered the Surface Markers of Splenic CD103^+^ DC and Polarization of Peripheral Blood Th Cells of Rats

**Figure 2:**
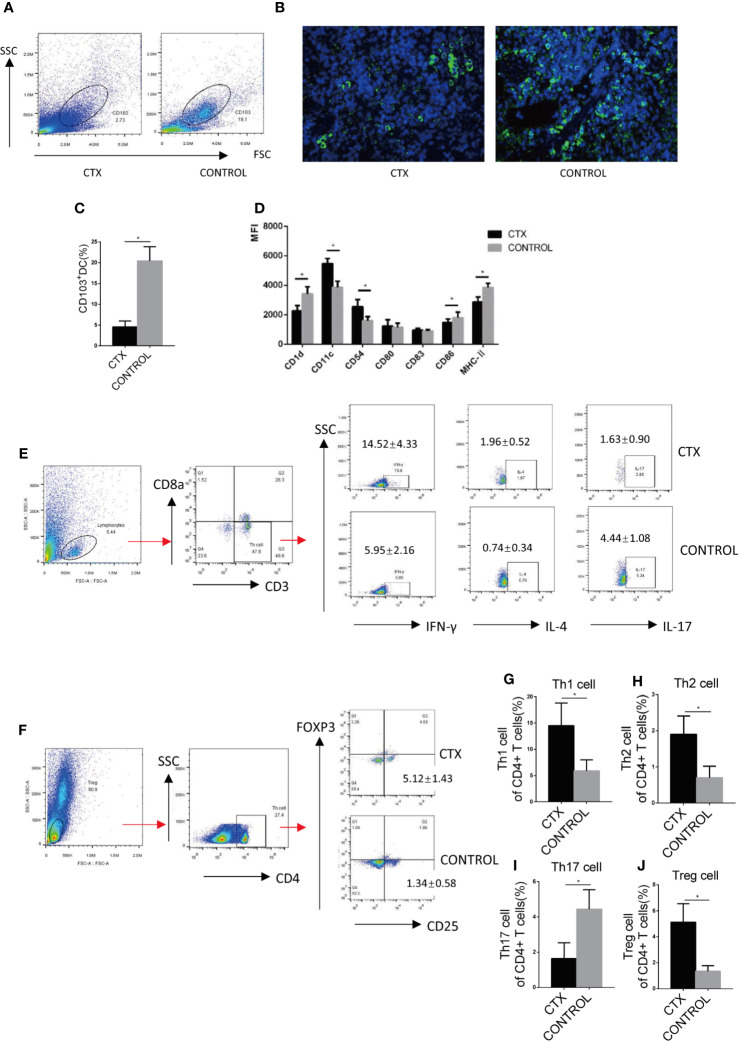
High-dose CTX altered the surface markers of splenic CD103^+^ DC and polarization of peripheral blood Th cells of rats. Splenic CD103^+^ DCs was determined by flow cytometry **(A)** and immunofluorescence **(B)**. **(C)** The quantitative results of splenic CD103^+^ DC. **(D)** Flow cytometric analysis of splenic CD103^+^DC surface markers. **(E)** Th cell polarization in the peripheral blood of rats. The quantitative results of Th1 **(G)**, Th2 **(H)**, Th17 **(I)**, and Treg **(J)** T cells in the peripheral blood of rats. n = 6. Error bars indicate the SEM. *P < 0.05, compared with the control group. All values represent the mean ± SEM.

We further examined splenic CD103^+^ DCs using flow cytometry and immunofluorescence. The number of CD103^+^ DCs isolated from the spleen was significantly reduced in CTX-treated rats relative to controls (*P <* 0.05; **Figures 2A–C**). CD103^+^ DCs surface markers were compared in the spleen of CTX-treated and untreated rats. The results showed that the median fluorescence intensity (MFI) values of CD11c and CD54 were increased and those of CD1d, CD86, and MHC-II were decreased in the spleen of the CTX-treated group (*P* < 0.05; **Figure 2D**). These results clearly demonstrated that high-dose CTX lowered CD103^+^ DC numbers, in addition to altering the expression of surface markers on this cell subset in the rat model. In addition, we detected the Th cells polarization in the peripheral blood of rats by flow cytometry. Our results revealed a significant increase in Th1, Th2, and Treg cell numbers, accompanied by a decrease in Th17 cells in peripheral blood of the CTX-treated rats, compared to the control group (*P* < 0.05; **Figures 2E–J**).

### High-Dose CTX Altered Antigen Uptake and the CD4^+^ T Cell Priming Capacity of imDC, As Well As Their Surface Marker Expression Profile *In Vitro*

We used a transcriptomic approach to detect the genetic alterations experienced by CTX-treated imDCs *in vitro* and found that 3,456 genes were up-regulated (**Figure S1A**). We found that the functions of the up-regulated genes were closely linked to metabolic processes (**Figure S1B**). Subsequently, in order to understand the mode of action of the up-regulated genes, we adopted the Kyoto Encyclopedia of Genes and Genomes (KEGG) analysis. KEGG analysis revealed the involvement of xenobiotic metabolism pathways and their regulation by cytochrome P450 (**Figure S1C**). Moreover, cytochrome P450 enzymes were included in those pathways (**Table S3**). These results demonstrated that imDCs may metabolize CTX *in vitro* by up-regulating the level of cytochrome P450 enzymes.

**Figure 3:**
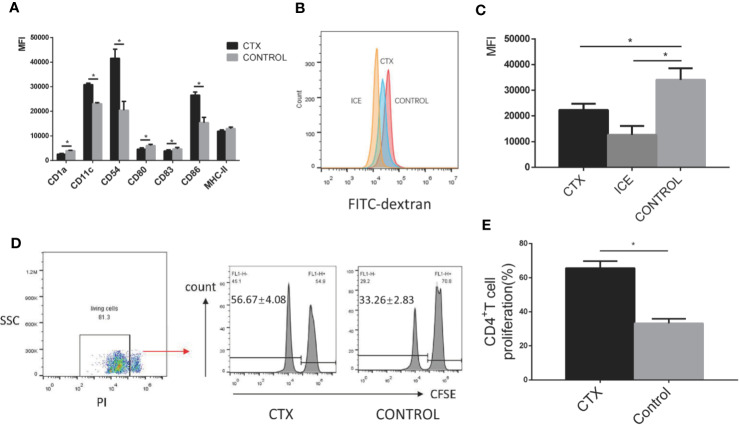
Alterations in the surface marker expression profile, antigen uptake and CD4^+^ T cell priming capacity of CTX-treated imDCs *in vitro*. **(A)** The quantitative results of imDC surface marker expression. **(B)** Detection of antigen uptake capacity of imDCs cultured at 37°C for 1 h in the presence of FITC-dextran by flow cytometry. **(C)** The quantitative result of antigen uptake capacity of CTX-treated imDCs. **(D)** The antigen presentation capacity of CTX-treated imDCs assessed by inducing CFSE-labeled CD4^+^ T cell proliferation in an MLR assay. **(E)** The quantitative result of antigen presentation capacity of CTX-treated imDCs. Dead cells were excluded from analysis based on PI fluorescence. Error bars indicate the SEM. *P < 0.05, compared with the control group. All values represent the mean ± SEM of three independent experiments.

To test the toxic effect of CTX on DCs *in vitro*, imDCs were treated with CTX for 24 h. According to our flow cytometry analyses, MFI values for the markers CD11c, CD54, and CD86 were increased, while those for CD1a, CD80, and CD83 were decreased in the CTX-treated samples, compared to controls (*P <* 0.05; **Figure 3A**). Furthermore, CTX-treated imDCs showed lower antigen uptake (*P <* 0.05; **Figures 3B, C**) and higher CD4^+^ T cell priming capacity, compared to untreated control samples (*P <* 0.05; **Figures 3D, E**). The results collectively demonstrated that high-dose CTX induced alterations in imDC surface marker expression profile, and a reduction in imDC antigen uptake capacity, while enhancing their ability to prime CD4^+^ T cells.

### High-Dose CTX Induced imDC-Mediated Th Cell Polarization

**Figure 4:**
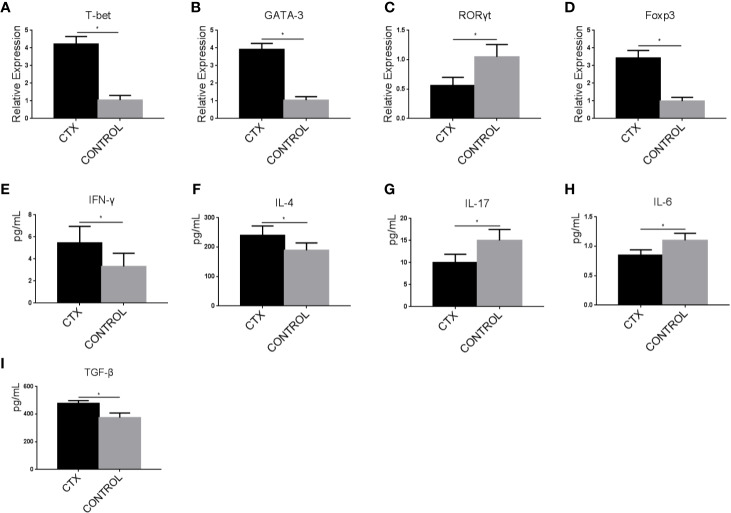
High-dose CTX induced imDC-mediated Th cell polarization. **(A–D)** RT-qPCR analysis of T-bet, GATA-3, RORγt, and Foxp3 mRNA levels in Th cells co-cultured with CTX-treated imDCs for 24 h. **(E–I)** ELISA detection of IFN-γ, IL-4, IL-17, IL-6, and TGF-β in Th cell/CTX-treated imDC co-culture supernatants. Error bars indicate the SEM. *P < 0.05, compared with the control group. All values represent the mean ± SEM of three independent experiments.

ImDCs have the unique ability to regulate immune responses through the activation and polarization of naïve T cells. Accordingly, we assessed T-bet, GATA-3, RORγt, and Foxp3 expression levels in Th cells co-cultured with CTX-treated imDCs by RT-qPCR. In the CTX-treated group, T-bet, GATA-3, and Foxp3 levels were significantly increased along with a concomitant reduction in RORγt expression, compared to the control group (*P < 0*.05; **Figures 4A–D**). ELISAs were further performed to evaluate the concentrations of IFN-γ, IL-4, IL-17, IL-6, and TGF-β in Th cells/CTX-treated imDC co-culture supernatants. Our data showed an increase in IFN-γ, IL-4, and TGF-β levels, accompanied by a decreased in IL-6 and IL-17 levels in the CTX-treated group, compared to the controls (*P* < 0.05; **Figures 4E–I**). Based on these findings, we concluded that in response to CTX, imDCs acted to significantly enhance Th1, Th2, and Treg cell-mediated immunity, while attenuating Th17 immunity, consistent with our *in vivo* data obtained from the immunosuppressive rat model.

### Enhanced Expression of TLR, MyD88, and MAPK Pathway-Associated Signaling Components in imDCs, in Response to CTX

**Figure 5:**
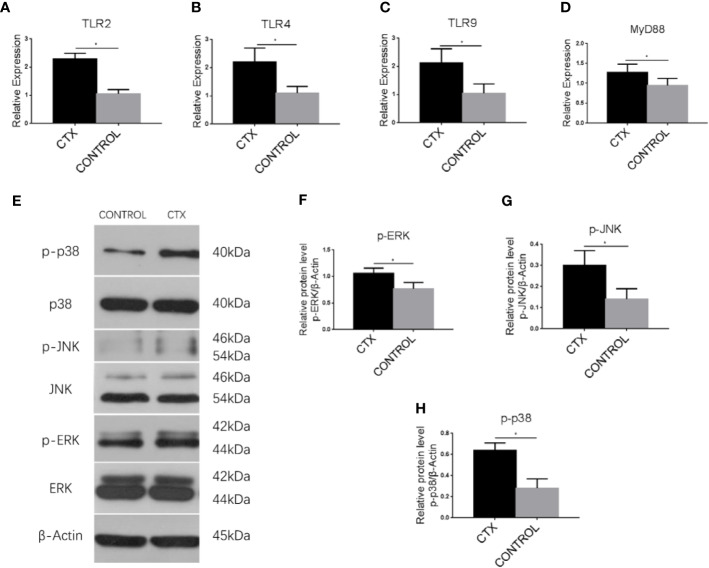
Enhanced expression of TLRs, MyD88, and MAPK pathway-associated signaling components in imDCs in response to CTX. **(A–D)** RT-qPCR analysis of TLR2, TLR4, TLR9, and MyD88 mRNA expression in imDCs. **(E–H)** Western blotting analysis of p38, p-p38, JNK, p-JNK, ERK, and p-ERK protein expression in imDCs. Error bars indicate the SEM. *P < 0.05, compared with the control group. All values represent the mean ± SEM of three independent experiments.

To establish the potential mechanism of Th cell polarization by imDCs, we conducted an RT-qPCR analysis of TLR2, TLR4, TLR9, and MyD88 mRNA. In addition, we performed western blotting to determine ERK, JNK, and p38 protein expression. Notably, TLR2, TLR4, TLR9, and MyD88 transcripts (*P <* 0.05; **Figures 5A–D**) and p-p38, p-JNK, and p-ERK protein levels (*P <* 0.05; **Figures 5E–H**) were significantly upregulated in the CTX-treated group.

## Discussion

In the present study, observation of morphology, organ coefficient, and proportion of T cells confirmed that the CTX-induced immunosuppressive rat model were established. In the rat model, we explored the splenic CD103^+^ DC numbers and found that a decrease in the number of splenic CD103^+^ DCs in response to CTX, indicating that CTX induced the apoptosis of CD103^+^ DCs. Subsequently, we found the expression patterns of antigen-presenting and co-stimulatory molecules on the surface of splenic CD103^+^ DCs were altered. These molecules have been identified as key factors in the induction of Th cell differentiation in the context of immunological diseases (Walsh and Mills, 2013; Cybulsky et al., 2016). Furthermore, high-dose CTX induces a reduction in the polarization of Th1/Th2 cells, consequently leading to the inhibition of the immune response (Wang et al., 2016). In the present study, we not only demonstrated the above phenomenon, but also detected the increase in Treg cell numbers accompanied by a drop in Th17 cells. There results suggested that splenic CD103^+^ DCs and Th polarization were affected by CTX in the rat model. Numerous studies have proved that chemicals and biomaterials can alter the phenotype and function of DCs and subsequently regulate Th cell polarization (Jeon et al., 2015; Park et al., 2015; Pattarini et al., 2017). Therefore, we give rise to the hypothesis that Th cell polarization in CTX-treated rats may be due to the effects exerted on DCs by high doses of CTX.

It is well-documented that CTX exerts its immunosuppressive effect once it is metabolized to phosphoramide mustard by the cytochrome P450 enzyme in the liver (Cartin-Ceba et al., 2017). The transcriptomic approach was used to detect the alterations of metabolic genes experienced by imDCs following CTX treatment *in vitro*. Our results showed that imDCs can metabolize CTX *in vitro* by up-regulating the expression cytochrome P450-associated genes. Subsequently, we detected the multiple surface markers of imDCs following CTX treatment *in vitro*, including the costimulatory proteins CD80 and CD86, the cell adhesion molecules CD11c and CD54, the activation marker CD83, and the antigen-presenting molecule MHC-II. Our results showed that the expression of those surface markers altered, as in the rat model. Those markers are in association with the antigen uptake and CD4^+^ T cell priming capacity of DCs (Diana et al., 2007). In addition, we assessed the antigen uptake and CD4^+^ T cell priming capacity of imDCs, which are the vital functions of DCs. The results demonstrated that CTX effectively reduced the antigen uptake capability of imDCs, in accordance with the action of other immunosuppressants (Holt and Thomas, 1997; Paolo et al., 2003). CTX enhanced the ability of imDCs to perform CD4^+^ T cell priming, which was in accordance with the findings of Holt (Holt and Thomas, 1997), who demonstrated that dexamethasone was ineffective in blocking the presentation of antigen to T cells in the MLR. Taken together, our data revealed that CTX altered the surface makers, as well as antigen uptake and CD4^+^ T cell priming capacity of imDCs, which were closely associated to polarization of Th cells.

DCs also provide key signals for polarization of Th cell subsets. T-bet, GATA-3, RORγt, and Foxp3 are the key transforming growth factors of Th1, Th2, Th17, and Treg cells, respectively (Yu et al., 2015). Furthermore, TGF-β can induce Tregs and also contribute to Th17 formation in the presence of IL-6 (Geginat et al., 2016; Ansa-Addo et al., 2017). We assessed T-bet, GATA-3, RORγt, and Foxp3 expression levels in Th cells co-cultured with CTX-treated imDCs. Our results showed that the polarization of Th cells altered: enhanced Th1 and Th2 responses, which were analogous to T-bet and GATA-3 expression. The Th17 response, represented by IL-17 and RORγt expression, decreased following CTX treatment. The Treg response increased in the CTX group. Moreover, increased TGF-β and lowered IL-6 levels promoted the differentiation of the Treg response and reduced the differentiation of the Th 17 response. In view of the finding that Th polarization is induced through MAPK signaling (Adler et al., 2007; Maria et al., 2009), we further evaluated several proteins associated with this pathway (such as p38, JNK, and ERK) in CTX-treated imDCs. Phosphorylation of these proteins was enhanced in CTX-treated imDCs, supporting the theory that CTX exerted its effect on Th cell polarization *via* the modulation of the MAPK pathway. Studies have found that DCs stimulate the Th1 response *via* IL-12 p70 secretion in a p38 MAPK phosphorylation-dependent manner (Jarnicki et al., 2008), Th2 cell polarization occurs *via* ERK phosphorylation (Agrawal et al., 2003; Kaisar et al., 2018). Moreover, p38 MAPK is proposed to be involved in the regulation of Th17 cells and Tregs (Cannon et al., 2013; Lu et al., 2014). We need to further explore the differential role of MAPK signaling of imDC in the polarization of Th cells.

In summary, CTX induced immunosuppression by reducing DC numbers and altering their surface marker expression profiles in our rat model. Additionally, CTX enhances antigen uptake, prime CD4^+^ T cells capacity and surface markers of imDCs *in vitro*, and altered DC-mediated Th cell polarization *via* the TLR/MyD88/MAPK pathway. It provides insights into the mechanisms implicated in the high-dose CTX treatment of imDCs may therefore be of great value in the management of autoimmune disease and transplantation.

## Data Availability Statement

The raw data supporting the conclusions of this article will be made available by the authors, without undue reservation, to any qualified researcher.

## Ethics Statement

The studies involving human participants were reviewed and approved by Ethics Committee of Zhengzhou University. The patients/participants provided their written informed consent to participate in this study. The animal study was reviewed and approved by the Institutional Animal Care and Use Committee of Zhengzhou University. Written informed consent was obtained from the individual(s) for the publication of any potentially identifiable images or data included in this article.

## Author Contributions

Conceptualization: LB and WY. Data curation: DW. Formal analysis: JW. Funding acquisition: WY. Methodology: CH, YZ, and YL. Project administration: WY. Writing—original draft: LB. All authors have read and agreed to the published version of the manuscript.

## Funding

This work was supported by the Natural Science Foundation of China (81273039).

## Conflict of Interest

The authors declare that the research was conducted in the absence of any commercial or financial relationships that could be construed as a potential conflict of interest.
