# Supplementary material for: High-Dose Cyclophosphamide Administration Orchestrates Phenotypic and Functional Alterations of Immature Dendritic Cells and Regulates Th Cell Polarization
Source: Front Pharmacol. 2020 May 25;11:775. doi: 10.3389/fphar.2020.00775 (PMC7261842; doi:10.3389/fphar.2020.00775)
Supplement: Supplementary file 1 [file DataSheet_1.docx]

Table S1 The primer sequences of genes

| Gene name | Forward primer | Reverse primer |
| --- | --- | --- |
| TLR2 | TTGTGACCGCAATGGTATCTG | GTTGTTGGACAGGTCAAGGCT |
| TLR4 | CAAGAACCTGGACCTGAGCTTTA | GATTTGTCTCCACAGCCACCAG |
| TLR9 | CGTGCAGCCGGAGATGTTT | CGTGAATGAGTGCTCGTGGTAG |
| MyD88 | GACCCAGCATTGAGGAGGATT | TCCTGCACAAACTGGATGTCG |
| T-bet | TGTGACCCAGATGATTGTGCTC | GTCACGGCAATGAACTGGGT |
| GATA-3 | CGAGATGGCACGGGACACTA | TGGTCTGGATGCCTTCCTTCTT |
| FoxP3 | CACTGACCAAGGCTTCATCTGT | TGTGGAGGAACTCTGGGAATGT |
| RORγ | GGCCATTCAGTACGTGG TGGAGTTCGC | CCGTGCGGTTGTCAGCATTGTAGGC |
| ACTION | CACCCAGCACAATGAAGATCAAGAT | CCAGTTTTTAAATCCTGAGTCAAGC |

Table S2 Weight and organ coefficient of CTX-exposed rat immunosuppressive model

| Group | Weight (g) | Spleen coefficient (%) | Thymus coefficient (%) |
| --- | --- | --- | --- |
| CTX | 266.400±31.437 | 0.123±0.041 | 0.068±0.006 |
| CONTROL | 341.400±26.006 | 0.198±0.383 | 0.185±0.039 |
| t | 4.110 | 3.031 | 6.579 |
| *p* | 0.003 | 0.016 | 0.002 |

Table S3 up-regulated genes of metabolism of xenobiotics by cytochrome P450 and drug metabolism-cytochrome P450

| Gene ID | Term | log2 | Qvalue |
| --- | --- | --- | --- |
| UGT2A1 | Metabolism;Human Diseases | 2.21519 | 3.63E-11 |
| GSTO2 | Metabolism;Human Diseases | 2.003858 | 8.44E-98 |
| ADH1C | Human Diseases;Metabolism | 3.014422 | 4.77E-06 |
| ADH4 | Metabolism;Human Diseases | 3.2043 | 9.54E-18 |
| CYP1A2 | Human Diseases;Metabolism | 2.611528 | 2.28E-110 |
| CYP2B6 | Metabolism | 2.714442 | 1.65E-27 |
| CYP2C18 | Metabolism;Organismal Systems;Human Diseases | 3.428961 | 0.000883 |
| CYP2F1 | Metabolism | 3.102923 | 9.04E-10 |
| CYP3A4 | Human Diseases;Metabolism | 2.324504 | 4.66E-05 |
| AKR1C1 | Metabolism;Organismal Systems | 3.515512 | 2.28E-135 |
| AKR1C2 | Metabolism;Organismal Systems | 1.790292 | 2.72E-16 |
| ALDH3A1 | Human Diseases;Metabolism | 1.643141 | 1.75E-14 |
| GSTM3 | Human Diseases;Metabolism | 1.521473 | 1.72E-103 |
| GSTT1 | Metabolism;Human Diseases | 5.577166 | 2.52E-07 |
| SULT2A1 | Metabolism;Human Diseases;Organismal Systems | 2.471543 | 3.16E-51 |
| UGT2B4 | Human Diseases;Metabolism | 3.218277 | 2.76E-13 |
| FMO1 | Metabolism | 4.294518 | 0.000672 |
| GSTT1 | Metabolism;Human Diseases | 5.577166 | 2.52E-07 |
| AOX1 | Environmental Information Processing;Metabolism | 2.930345 | 0.000304 |
| UGT2B15 | Metabolism;Human Diseases | 3.97828 | 1.32E-27 |


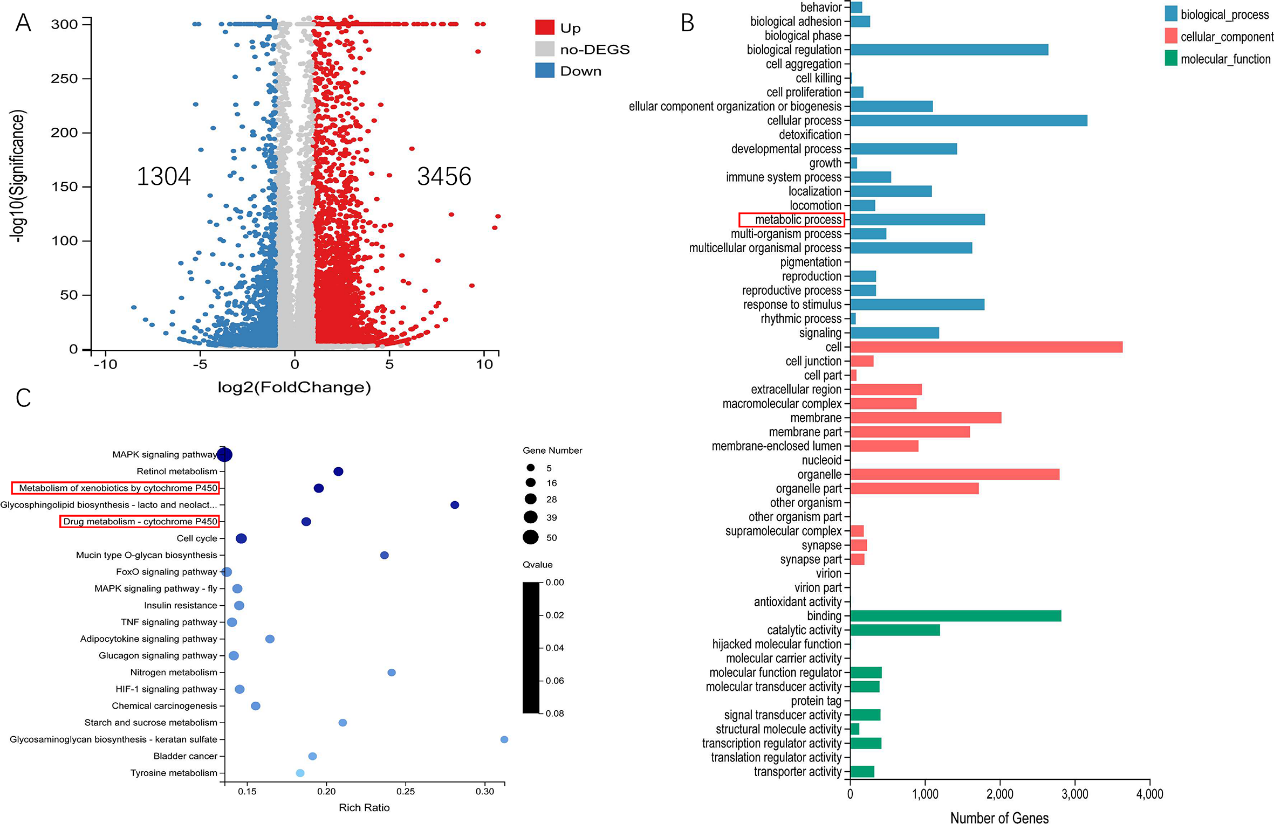


Fig.S1 DCs can metabolize CTX in vitro by up-regulating the level of cytochrome P450. (A) The screening results of differently expressed genes. (B) Gene ontology analysis of up-regulated genes. (C) KEGG pathway enrichment analysis of up-regulated genes.
